# ASO Author Reflections: Investigating Social Risk Factors for Melanoma Nodal Surveillance Adherence in Real-World Populations

**DOI:** 10.1245/s10434-024-16683-x

**Published:** 2024-12-09

**Authors:** Kelsey B. Montgomery, Kristy K. Broman

**Affiliations:** 1 Department of Surgery, University of Alabama at Birmingham, Birmingham, AL USA; 2 Institute for Cancer Outcomes and Survivorship, University of Alabama at Birmingham, Birmingham, AL USA; 3 Birmingham Veterans Affairs Medical Center, Birmingham, AL USA

## Past

The Second Multicenter Selective Lymphadenectomy Trial (MSLT-II) changed management of sentinel node positive (SLN+) melanoma, with the majority of SLN+ patients now receiving nodal surveillance rather than completion lymphadenectomy.^1,2^ The MSLT-II nodal surveillance protocol requires frequent visits for nodal ultrasound (US) and examinations, which may present barriers to patients with social risk factors, such as lack-of or under-insurance, or limited transportation.^3,4^ Investigation of the potential impacts of social determinants of health (SDoH) is needed to understand and address potential barriers to melanoma nodal surveillance.

## Present

This study evaluated the association between individual- and area-level SDoH on melanoma nodal surveillance US adherence, combined modality adherence, and loss to follow-up (LTFU).^5^ No statistically significant differences in US adherence were seen based on SDoH covariates, but increased likelihood of LTFU was seen among Medicaid and uninsured patients, suggesting that individual-level SDoH may impede consistent nodal surveillance. Importantly, the overall rate of US adherence was 41.6% (range 7.7–73.7% across centers) and the overall LTFU rate was 29.1% (range 0.0–60.2%). Beyond patient social risk factors, these wide ranges in US adherence and LTFU likely reflect an early post-MSLT-II implementation period, as well as variation in surveillance practices across providers and institutions.

## Future

Moving forward, the prospective collection of individual-level SDoH data may provide more granularity about specific social risk factors that can affect adherence to nodal surveillance. This information could be used to support shared decision making about the appropriateness of nodal surveillance versus completion lymphadenectomy and help institutions identify at-risk individuals who can benefit from supportive services such as financial counseling or transportation assistance. While risk factors may vary across geographical regions or rural/urban settings, collaboration across melanoma centers to share effective strategies for social risk factor mitigation could provide insights for other institutions managing similar barriers.
